# Comments on Foucher's flap

**DOI:** 10.4103/0970-0358.59300

**Published:** 2009

**Authors:** B. Jagannath Kamath

**Affiliations:** Department of Orthopaedics, Kasturba Medical College, Mangalore, Karnataka, India. E-mail: bjkamath@satyam.net

Foucher's flap is one of the ideal flaps to cover traumatic defect of the pulp and dorsum of the thumb. Though ideally described for thumb defects with some shortening, it has been proved beyond doubt that it can also be requisitioned to resurface thumb defects without shortening. In the latter indication, it is necessary to harvest the flap up to the dorsum of the PIP joint. However, the distal 1/3 of such a first dorsal metacarpal artery (FDMA) flap becomes a random part of the axial artery flap and hence carries some risk of marginal necrosis. It is surmised that the survival of such a flap is ultimately determined by the relative length of index and thumb.

Normally, the tip of the adducted thumb roughly reaches upto 70% of the length of the proximal phalanx of the index finger (32% of the index finger length beyond MP joint);[1,2] such thumbs being ideally suited for conventional FDMA flaps. Individuals with thumb length more than 70% of the proximal phalanx of index finger are the ones likely to need an extended FDMA flap i.e. wherein flap is harvested beyond the PIP joint. One more ambiguous parameter of this flap is the amount of blood supply which comes alongwith the superficial branch of radial nerve. We know that every superficial nerve is accompanied by an artery and vein of its own supplying the skin and integument. Being a neuro-sensory flap, it is an ideal flap for pulp of thumb which restores an acceptable level of sensation, however, it may not match Littler's neuro-vascular island flap because of poorer cortical reorientation.

Kulkarni *et al.*[3] need to be congratulated for describing one more indication for FDMA flap. Though small traumatic defects on the radial side of the palm are rare, when encountered, this flap is really handy when compared to the alternatives. These flaps are also used in reconstructive surgeries following post burn scar contracture release,[4] syndactyly release, and partial loss of reimplanted thumb.[5] Donor-defect following this flap usually needs split skin graft or full-thickness skin graft. These usually do not give rise to functional deficit. Cosmesis, however, will be a consideration. This can be avoided if one harvests only adipofascial flap to cover the thumb defect as described by Vishwa Prakash.[6] One more modification suggested is the usage of this flap as distally-based flap for coverage of web and ulnar aspect of the dorsum of the hand and distal dorsum of the index. The size of the FDMA flap at widest portion could be 1.2 to 1.5mm which for some super micro surgeons may be an option for a small free neuro-vascular flap for other digits.

The article gives a lucid description and illustration of the use of the FDMA flap for thumb defects. However, I feel it may be risky to perform this flap without loop magnification, under local anaesthesia and without proper tourniquet. The chances of survival can be further enhanced by including more than one dorsal vein as described by El Khatib,[7] who has harvested the flap from not only the dorsum of proximal but also from the middle phalanx of the index finger extending into what he calls the “dynamic territory” or the random part of the flap. He argues in favour of including the dorsal veins to prevent venous congestion in the flap. His modification enables the surgeon to cover any defect on the thumb of normal length. The same philosophy has been further propagated by Gebhard *et al*.[8] in his case report wherein he has harvested the flap up to the DIP joint so as to wrap around the proximal phalanx of a traumatized but shortened thumb which was found unsuitable for reimplantation. If one wants to play safe and use this extended FDMA flap, it may not be a bad idea to delay this distal dynamic territory/random part of the flap in the first stage and raise the whole of the extended FDMA flap in the second stage.

The arterial pedicle of the flap, which is the ulnar-most of the three branches, coming from radial artery just before dipping in between the two heads of first dorsal interossei is almost constant and is amenable to be pivoted as proximally as the medial border of the EPL in line with the radial border of the second metacarpal. This unique arterial supply gives the surgeon a wide arc of rotation with the pedicle length of 6-7 cm in adults; as in the present report.
